# Cognitive test performance in chronic cannabis flower users, concentrate users, and non-users

**DOI:** 10.1038/s41598-023-35217-1

**Published:** 2023-05-18

**Authors:** Carrie Cuttler, Aria S. Petrucci, Emily M. LaFrance

**Affiliations:** Department of Psychology, Washington State University, PO Box 644820, Pullman, WA 99164-4820 USA

**Keywords:** Drug safety, Human behaviour

## Abstract

Extremely high-potency cannabis concentrates are becoming increasingly available and popular among consumers. While prior research indicates these products are perceived to have greater detrimental effects relative to cannabis flower, few studies have examined their relative objective effects, and no existing studies have compared the cognitive test performance of sober flower users, concentrate users, and non-users. A total of 198 healthy adults (98 non-users, 46 exclusive flower users, and 54 concentrate users) were administered a battery of tests of memory, psychomotor speed, attention, and executive functioning under sober laboratory-controlled conditions. Significant group differences were detected on tests of verbal free recall and episodic prospective memory, with both the flower users and concentrate users demonstrating significantly worse performance than non-users. Concentrate (but not flower) users performed worse than non-users on a measure of source memory, but contrary to our hypothesis, there were no significant differences between flower and concentrate users on any of the cognitive tests. Results indicate that, under sober conditions, individuals who regularly use concentrates are no more cognitively impacted than those who exclusively use flower. These null findings may reflect the tendency for concentrate users to self-titrate and use significantly lower quantities of concentrates than flower.

## Introduction

Recent trends towards state-level cannabis legalization have facilitated substantial changes in the cannabis industry, namely the proliferation of different types of cannabis products available for purchase. Notably, cannabis concentrates (also known as extracts, wax, shatter, budder, hash/butane oil) are a novel and highly potent form of cannabis gaining popularity among consumers. Concentrates are typically produced via use of chemical solvents (e.g., butane) or a process of agitation and filtration to extract very high concentrations of delta-9-tetrahydrocannabinol (THC)—the primary psychoactive constituent of cannabis—from the cannabis plant^1,2^. Typically, the end-product is an oil, wax, or resin-like substance which is consumed by inhaling the vapor of the heated concentrates (e.g., dabbing). Although flower typically contains approximately 9–20% THC^3–6^ and more rarely exceeds 30% THC, the average THC content of concentrates typically exceed 60%^2,5,6^ and can exceed 90%^4,7^. These extremely high-potency products are becoming increasingly popular, with recent research indicating that 58–66%^8–10^ of adult cannabis users have used concentrates, and 13–37% use them on a regular basis^8,9^. Moreover, analysis of the Washington state cannabis market revealed that cannabis concentrate shares increased by 146% from 2014 to 2016, while flower shares decreased by 22%^5^, trends which are expected to continue into the future^11^.

Despite their widespread availability, popularity, and potency, relatively little research has examined the effects of concentrate use compared to flower use. This is in part because the federal classification of cannabis as a Schedule 1 drug has historically barred researchers from accessing cannabis concentrates. The novelty and range of concentrate products, and their various extraction methods, also make this research challenging. Nevertheless, evidence suggests that negative outcomes associated with cannabis increase with dose and potency. For instance, higher potency cannabis flower and higher doses of THC are associated with more severe cannabis dependence^12^, increased risk of psychosis^13,14^, and worse cognitive performance^15,16^. Although findings from naturalistic studies suggest that cannabis users self-titrate by taking fewer puffs of concentrates than flower^17^ and report equivalent levels of intoxication after use^17,18^, higher plasma levels of THC and cannabis metabolites have been detected in concentrate users^18^. As such, negative outcomes associated with cannabis use may be exaggerated by use of concentrates due to increased THC exposure.

This sentiment is echoed by cannabis users’ perceptions of the risks associated with concentrate use and is provisionally supported by the limited literature exploring detrimental outcomes associated with concentrate use. Cannabis users perceive concentrates to be riskier than flower^9,19^, and both concentrate users and non-users perceive greater risk of developing addiction, depression, anxiety, and psychosis due to the use of concentrates versus flower^8^. Consistent with these perceptions, concentrate users are more likely to ever receive a diagnosis of a psychological disorder, including depression and anxiety, than those who use other forms of cannabis^20,21^. Relative to flower, concentrate use has also been suggested to be associated with increased frequency of cannabis use^20,22^, younger age of onset of cannabis use^23^, tolerance and withdrawal^19^, symptoms of cannabis use disorder^7^, physical dependence^24^, risky behavior^23^, as well as use of other drugs^20,23^. It is important to note that most of these studies are cross-sectional and therefore, with the exception of age of onset of cannabis use, it is difficult to know whether concentrate use increases the risk of these outcomes or whether these outcomes increase the likelihood that individuals will use concentrates. Nevertheless, taken together, these findings imply that concentrate use is associated with more negative outcomes than use of lower potency forms of cannabis, although the direction of these associations remains unclear.

However, the cognitive effects of concentrate use, relative to flower use, have scarcely been examined. In general, acute and chronic cannabis use is associated with diminished cognitive test performance, and these effects have most reliably been detected using tests of memory, particularly verbal memory^25,26^. Decreased performance on executive functioning, psychomotor functioning, processing speed, attention, and decision-making tests have also been observed in chronic cannabis users with effect sizes generally ranging from small-to-moderate^26,27^. We note, however, that many of these effects were observed in frequent cannabis users. Given evidence that frequency of chronic cannabis use is inversely associated with performance on many cognitive tasks^25^, some effects may be weaker or absent in infrequent cannabis users, and impairments outside the domain of memory are generally less reliable even in frequent users^26^. Tenuously supporting the possibility that concentrates may compound these effects, concentrate users perceive greater risk of developing problems with memory, concentration, and motivation from concentrate than flower use^8^. Similarly, cannabis users subjectively report poorer memory and attention^20^, as well as decreased cognitive enhancement (e.g., fewer ideas, less motivation, etc.) but paradoxically also lower cognitive impairment^22^ when using concentrates versus flower.

To our knowledge, only three prior studies have objectively compared the cognitive effects of flower versus concentrate use. In one study, investigators found a main effect of product type on inhibitory control, such that concentrate users performed worse than flower users (collapsed across assessments before and after using cannabis) on the NIH Toolbox Flanker Inhibitory Control and Attention Test^18^. However, the interpretability of this finding is somewhat limited because both groups’ task performance improved from pre- to post- cannabis use (likely due to practice effects). Concentrate and flower users did not differ at baseline or after cannabis use on measures of memory, including the NIH Toolbox Picture Sequence Memory Test (which assesses episodic memory), the NIH Toolbox List Sorting Working Memory Test, the International Shopping List Task (which assesses verbal free recall), or physical balance (with eyes open, eyes closed, and eyes closed and head tilted). Similarly, an extension of this study found comparable acute effects of flower and concentrate use on measures of balance and reaction time from the DRUID mobile app which was designed to assess impairment^28^.

A third naturalistic study again revealed no differences in the acute effects of concentrates and flower on cognition^17^. In this study, chronic cannabis users (experienced using both flower and concentrates) were randomly assigned to inhale either concentrates (> 60% THC) or high-potency flower (> 20% THC, with or without cannabidiol [CBD]) or remain sober prior to completing a series of cognitive tests remotely via Zoom. The cognitive test battery comprised several measures of memory (including two measures of prospective memory, a source memory test, a temporal order memory test, and the Deese-Roediger-McDermott False Memory Paradigm) and non-normative decision-making (specifically the Under/Overconfidence Test of Metacognition, Resistance to Framing Test, Consistency in Risk Perception Test, and Resistance to Sunk Cost Test). Relative to the sober control group, both the flower and concentrate groups had worse source memory for pictures (i.e., they were more likely to incorrectly report that items they saw as pictures were presented as words or not presented at all). Both the flower and concentrate groups also demonstrated an increased susceptibility to false memories for unrelated words (i.e., they had more false alarms for words that were completed unrelated to word lists they studied), relative to the sober control group. The concentrate group further demonstrated increased susceptibility to false memories for related words (i.e., false alarms for words related to word lists they studied) relative to the sober control group. Surprisingly, the group that used flower containing both THC and CBD had significantly worse free recall of pictures relative to all other groups, including the concentrates group. The concentrates group did not perform significantly worse than the flower groups on any cognitive outcomes. Clearly, more research is needed to further examine whether concentrates and flower differentially affect cognition, as suggested by subjective reports that remain uncorroborated by objective studies. Moreover, research involving a sober, non-user control group is important to appropriately address the potential long-term cognitive effects of concentrates.

Accordingly, the objective of the present study was to examine sober cognitive test performance in adult non-users, exclusive flower users, and concentrate users. To this end, we recruited a sample of non-users and cannabis users to complete a measure of cannabis use patterns (including types of products used) and a battery of tests of memory, attention, psychomotor speed, and executive functioning. These domains of cognition were selected because they have revealed effects of chronic cannabis use in prior research^25–27^. We included more measures of memory than other cognitive constructs since this domain of cognition appears to be the most reliably impacted by chronic cannabis use^26^ but impacts of chronic use of cannabis on aspects of memory other than verbal memory and working memory have been under investigated (e.g., prospective memory) or not investigated at all (e.g., source memory, temporal order memory). The inclusion of a non-using control group allows us to determine whether there are significant differences between concentrate users and/or flower users relative to non-users, comparisons which are crucial for examining potential impairments from chronic cannabis use as well as for later interpreting any observed differences between concentrate and flower users. We hypothesized that both flower and concentrate users would demonstrate diminished cognitive test performance relative to non-users, and, further, that concentrate users would perform worse than flower users. Consistent with the prior literature, we expected to find more robust detrimental effects of chronic cannabis use on memory (particularly verbal free recall), relative to the other domains of cognition assessed.

## Methods

### Inclusion/exclusion criteria

Eligible participants had to be adults between the ages of 18 and 39 to reduce effects of age-related cognitive decline, which begins in middle and older adulthood^29–31^. Participants were not eligible if they had any diagnosed psychiatric, neurological, or serious medical conditions; reported severe depression (score > 20), anxiety (score > 14), or stress (score > 20) on the Depression, Anxiety, Stress Scales (DASS-21)^32^; were taking prescription medication; used any illicit drugs in the past 6 months; reported heavy drinking (> 4 drinks > 4 times/week) or heavy smoking (> 20 cigarettes/day); or were not fluent in English (< 5 years speaking English).

To be considered a non-user, participants had to report fewer than 10 lifetime uses, no use in the past year, and have a negative urine test for THC. To be considered a cannabis user (flower or concentrate user), they had to report using cannabis on a daily or near daily basis (> 3 times per week) for at least 1 year and have a urine test positive for THC. Flower users had to report exclusive use of flower and concentrate users had to report using concentrates at least 25% of the time they use cannabis. Further, cannabis users had to abstain from using any cannabis products on the day of the testing session (before midnight). A small number of participants (*n* = 6) who reported using cannabis on the day of testing were excluded from analyses. The vast majority of the cannabis users (96%) reported last using cannabis the day prior to the testing session (the remainder reported last using cannabis earlier that week). Participants were screened for most eligibility criteria prior to scheduling their testing session (except for the DASS-21, urine test, and last time they used cannabis) and those deemed eligible were screened a second time at the end of their testing session to reconfirm their eligibility.

### Participants

Participants were recruited from the local community using flyers and advertisements on social media. A power analysis indicated that a sample size of 189 would afford power of 0.80 to detect small-to-moderate sized effects (η_p_^2^ = 0.05). Consistent with this, a sample of 98 non-users and 100 cannabis users met the eligibility criteria and were included in analyses. Of the 100 cannabis users, 46 reported exclusive use of flower, 1 reported exclusive use of concentrates, and 53 reported regular use of both concentrates and flower. Regular use was defined as use of the product at least 25% of the time they use cannabis. The 46 exclusive flower users comprise the flower user group and the 54 regular users of concentrates comprise the concentrate user group.

Most cannabis users (88%) reported using cannabis at least once per day, and approximately half (47.5%) reported first trying cannabis before the age of 16. Flower users reported using a mean of 0.75 g (*SD* = 0.68) of flower per day, a mean of 3.15 (*SD* = 2.63) flower sessions per day, and a mean total duration of use of 8.13 years (*SD* = 6.05). Concentrate users reported taking a mean of 6.24 hits/dabs (*SD* = 11.15) of concentrates per day, a mean of 3.63 (*SD* = 6.85) concentrates sessions per day, a mean of 3.77 (*SD* = 3.04) flower sessions per day, and mean total duration of use of 6.65 years (*SD* = 4.35). As shown in Table 1, the three groups (non-users, flower users, concentrate users) were well matched with respect to age, gender, ethnicity, personal income, premorbid verbal IQ, depression, and stress. However, the two groups of cannabis users (flower users and concentrate users) reported significantly lower education, higher anxiety, and higher scores on the Alcohol Use Disorders Identification Test (AUDIT)^33^ than did the non-users. As such, education, anxiety, and AUDIT scores were included as covariates in all subsequent analyses. The two groups of cannabis users did not differ significantly with respect to any demographic characteristics, depression, anxiety, stress, AUDIT scores, daily cannabis use sessions, flower sessions per day, frequency of cannabis use, age of onset of cannabis use, or quantity of edibles typically used (see Table 1). However, concentrate users reported using significantly higher quantities of cannabis flower than did flower users. Nevertheless, the two groups had comparable THC urine test results.

**Table 1:** Demographic characteristics and cannabis use patterns.

	Non-users (*n* = 98)	Flower users (*n* = 46)	Concentrate users (*n* = 54)	Group comparisons
Gender	%	%	%	${\chi }^{2}$= 3.68, *p* = 0.45, Φ = 0.14
Men	45.9	50.0	46.3	
Women	54.1	47.8	53.7	
Non-binary	0	2.2	0	
Ethnicity	%	%	%	${\chi }^{2}$ = 4.80, *p* = 0.09, Φ = 0.16
White	58.2	76.1	68.5	
Education	%	%	%	${{\varvec{\chi}}}^{2}$** = 33.52, *p* = 0.002, Φ = 0.41**
Less than high school	0	2.2	0	
High school or equivalent	4.1	10.9	20.4	
Some college (no degree)	38.8	52.2	53.7	
Trade/technical/vocational	1.0	2.2	0	
Associate degree	10.2	13.0	9.3	
Bachelor’s degree	33.7	10.9	13.0	
Master’s degree	10.2	8.7	0	
Doctorate degree	2.0	0	3.7	
Age	Mean (*SD)*	Mean (*SD)*	Mean (*SD)*	*F* = 2.46, *p* = 0.09, $\upeta$_p_^2^ = 0.02
	24.01 (4.65)	23.96 (4.50)	22.48 (3.24)	
Personal income	Mean (*SD)*	Mean (*SD)*	Mean (*SD)*	
	1.79 (1.16)	1.74 (0.93)	1.62 (1.00)	*F* = 0.40, *p* = 0.67, $\upeta$_p_^2^ = 0.004
Premorbid verbal IQ	Mean (*SD)*	Mean (*SD)*	Mean (*SD)*	
	39.43 (6.89)	37.05 (6.65)	36.84 (6.95)	*F* = 1.20, *p* = 0.30, $\upeta$_p_^2^ = 0.01
DASS scores	Mean (*SD)*	Mean (*SD)*	Mean (*SD)*	
Depression	3.02 (3.75)	4.09 (4.60)	3.33 (4.23)	*F* = 1.06, *p* = 0.35, $\upeta$_p_^2^ = 0.01
Anxiety	2.98 (3.33)^a^	6.36 (3.79)^b^	6.34 (3.72)^b^	***F* = 21.78, *p* < 0.001, **${\varvec{\upeta}}$_**p**_^**2**^** = 0.19**
Stress	5.57 (5.68)	7.48 (5.83)	7.37 (5.55)	*F* = 2.51, *p* = 0.08, $\upeta$_p_^2^ = 0.03
AUDIT scores	Mean (*SD)*	Mean (*SD)*	Mean (*SD)*	***F* = 17.92, *p* < 0.001, **${\varvec{\upeta}}$_**p**_^**2**^** = 0.17**
	3.18 (2.28)	5.75 (3.62)	6.39 (4.07)	
DFAQ-CU *z* scores	Mean (*SD)*	Mean *(SD)*	Mean (*SD)*	
Daily sessions	–	− 0.12 (0.92)	0.05 (0.97)	*t* = − 0.87, *p* = 0.39, *d* = 0.17
Frequency	–	− 0.10 (0.58)	0.08 (0.68)	*t* = − 1.37, *p* = 0.18, *d* = 0.27
Age of onset	–	0.03 (0.49)	− 0.04 (0.49)	*t* = − 0.70, *p* = 0.48, *d* = 0.14
Flower quantity	–	− 0.22 (0.45)	0.18 (1.08)	***t* = **− **2.35, *p* = 0.02, *d* = 0.47**
Edibles quantity	–	− 0.21 (0.69)	0.11 (1.14)	*t* = − 0.84, *p* = 0.41, *d* = 0.32
THC (urine)				
	0.00 (0.00)	4.09 (1.11)	4.09 (1.10)	*F* = 671.66, p < 0.001, $\upeta$_p_^2^ = 0.87

Bolded statistics represent significant group differences. Personal income was measured using the following scale: 1 =  < $10K, 2 = $10–20K, 3 = $20–40K, 4 = $40–60K, 5 = $60–80K, 6 = $80–100K, 7 =  > $100K. THC measured as 0 = 0 ng/ml, 1 = 25 ng/ml, 2 = 50 ng/ml, 3 = 150 ng/ml, 4 = 300 ng/ml, 5 = 600. ng/ml. AUDIT scores below 8 suggest low risk alcohol consumption according to World Health Organization guidelines. DFAQ-CU scores are displayed as *z* scores, consistent with the published scoring criteria^46^.

### Procedure

The study was approved by the Washington State University Institutional Review Board and adhered to the Declaration of Helsinki. All participants were screened to determine eligibility prior to enrollment and a second time after cognitive testing (after they had earned their compensation and had diminished motivation to deceive us). Participants who passed the initial screening but disclosed information that made them ineligible were compensated, but their data were excluded from analyses.

Participants provided informed consent prior to completing the cognitive tests and questionnaires described in the “Materials” section. All tests were completed one-on-one with experimenters trained in the proper administration of cognitive tests and blinded to participants’ cannabis-using status. Following the completion of these tests, participants were given a computer to complete an online survey containing the questionnaires described in the “Materials” section. The testing session took place in a quiet controlled laboratory environment. At the end of the testing session, participants provided a urine sample, and a 6-level commercially available THC test (NarcoCheck) was used to confirm cannabis user status via the presence or absence of THC. This test was administered at the end of the testing session so the experimenter would remain blind to their cannabis-using status until after testing was complete. The entire testing session took approximately 2 h and participants were compensated with $50 for their time.

### Materials

#### Weschler Test of Adult Reading (WTAR)

The WTAR is a brief measure of premorbid verbal IQ^34^. The test consists of a list of 50 words that participants were asked to pronounce aloud. They were given 1 point for each correctly pronounced word. This test was used to ensure groups had comparable premorbid verbal IQ which could impact cognitive test performance.

#### Prospective memory tests

Participants’ ability to remember to execute tasks in the future was assessed with two prospective memory tests. To assess episodic prospective memory, participants were asked to remember to request their $50 compensation before they began the online questionnaires that were administered at the end of the study (Episodic Prospective Memory)^17,35–37^. Performance was scored in a binary manner with participants who successfully remembered to request their compensation at the appropriate time receiving a score of 1 and those who failed to do so receiving a score of 0.

To assess habitual prospective memory, participants were asked to try to remember to spontaneously rate how difficult they found each test immediately after completing each one (Habitual Prospective Memory)^17^. Performance was scored by assigning 1 point per test for which the participant successfully provided a difficulty rating.

#### California Verbal Learning Test-II (CVLT-II)

Verbal free recall was assessed using an abbreviated version of the CVLT-II^38^. For this test, participants were asked to listen to and immediately recall a list of 16 words, three times in a row, and then to listen to and immediately recall a new list of 16 words one time. The mean number of words recalled across these 4 trials was used as an index of immediate verbal memory (Verbal Memory–Immediate). Immediate verbal memory scores can therefore range from 0 to 16. Approximately 20 min later, participants were asked to recall the words from the original list without re-presentation of the list. The number of words correctly recalled was used as an index of delayed verbal memory (Verbal Memory–Delayed). Scores can range from 0 to 16 on this trial, with higher scores indicating better verbal memory.

#### Brief Visuospatial Memory Test-Revised (BVMT-R)

The BVMT-R was used to assess visuospatial memory^39^. For this test, participants were shown an array of 6 geometric figures for 10 s and then were asked to draw as many of the figures as they could in their correct location on the page. This trial was repeated a second time. They were given 1 point for each correct figure they drew and an additional point if the figure was in its correct location (for a total of 12 points per trial). Scores on these two trials were averaged to derive the index of immediate visuospatial memory (Visuospatial Memory–Immediate). As such, scores can range from 0 to 12, with higher scores reflecting better visuospatial memory. Participants were also asked to reproduce the figures in their correct locations 20 min after the second immediate recall trial (without re-presentation of the figures) for an index of delayed visuospatial memory (Visuospatial Memory–Delayed). Scores on this trial could also range from 0 to 12.

#### Source memory test

To assess source memory^40^, participants were first shown 32 basic pictures of common objects (e.g., line drawing of a hat) and printed words for 2-s each. After approximately 10 min, participants freely recalled as many items as possible. The number of correctly recalled items was computed as an index of free recall of pictures and words (Free Recall–Pictures/Words). Scores on this index could range from 0 to 32 which higher scores indicating better free recall. Next, participants were presented with 64 words and were asked to identify whether each was presented previously as a picture or a word or whether the item was not presented earlier. The number of correctly recognized items was computed as an index of source memory (Source Memory). Scores on this index could range from 0 to 64 with higher scores indicating better source memory.

#### Temporal- order memory test

After completing all other tests, participants were asked to recall all the tests they completed (in any order) which serves as an index of episodic recall (Episodic Recall)^40^. Next, participants were given a set of cards describing each test and were asked to organize them in the order in which they were completed^40^. Tests placed in the correct sequence were given 2 points each. If they switched the order of a test with an adjacent test or recalled a series of tests in the correct order, but in the incorrect location within the larger list, they received scores of 1 for each test. Tests recalled completely out of sequence were assigned a score of 0. Scores were then summed to assess temporal-order memory (Temporal-Order Memory).

#### Digit Span Backward Test (DSB)

The DSB was administered as a measure of working memory^41^. For this test, participants hear sequences of numbers and repeat/recall each in reverse order. The number of digits in the sequence begins with 2 and increases one at a time up to a length of 8 numbers. The test includes two sequences of each length and testing ceases if a participant fails to correctly recall any two sequences of the same length. The total number of sequences successfully recalled was recorded and served as our index of working memory (Working Memory). Possible scores range from 0 to 14.

#### Digit Symbol Substitution Test (DSST)

The DSST is a standardized measure of psychomotor speed and attention^42^. Participants were given a sheet of paper displaying a row of digits, each paired with a unique symbol, and 7 additional rows of digits each paired with an empty box. Participants were required to fill in the empty boxes by writing the corresponding missing symbol—the symbol that is paired with each digit in the first row—as quickly and as accurately as possible. The number of correct symbols entered in 90 s was used as an index of psychomotor speed/attention (DSST–Copy Trial). Scores on this trial can range from 0 to 113, with higher scores indicating better psychomotor speed/attention. The form was then folded so that participants could no longer see the legend of digits and symbols and they were asked to write the correct symbol in the last row of empty boxes using their memory instead of the legend. This trial was used as an index of incidental recall (Incidental Recall). The last row contained 20 boxes so scores on the incidental recall trial could range from 0 to 20.

#### Ruff 2 & 7 Selective Attention Test (Ruff 2 & 7)

The Ruff 2 & 7 Selective Attention Test^43^ was also administered to measure attention. For this test, participants were given 2 sheets of paper, each containing 30 rows of 50 numbers and letters divided into 10 sections (each section had 3 rows of 50 numbers and letters). Participants were instructed to cross out every 2 and 7 they see, working left to right one row at a time. They were given 15 s for each section and then were instructed to move onto the next section for a total of 20 sections. The total number of 2 s and 7 s correctly crossed out was tallied as an index of attention (Selective Attention). There were 30 targets (2 s and 7 s) in each of the 20 sections so possible scores can range from 0 to 600.

#### Zoo Map Test

The Zoo Map Test^44^ was used to measure executive functioning, specifically planning. For this test participants were shown a map of a zoo and were asked to draw a route around the zoo to visit all the places listed in a set of instructions, while following a set of rules. For the first trial, they were asked to start at the entrance and finish with a picnic. They were told they can use shaded paths as many times as they would like but can only use unshaded paths once. There is a camel ride they can use to travel but they could only use it once. Following this initial trial, they were then given a new map and were asked to repeat this process while following additional rules requiring them to visit specific places in certain orders (e.g., they must visit a café after visiting elephants). We recorded correct paths and errors and derived profile scores that can range from 0 to 4, using the published scoring manual (Zoo Map Test Profile Score). Higher profile scores indicate better executive functioning.

#### Tower Test

We also administered the Tower Test^45^ as a second index of the planning component of executive functioning. For this test, participants were given a wooden base with 3 pegs and wooden disks of various sizes. The disks were first positioned on the pegs by the experimenter and then participants were asked to move the discs to build towers that look like specific pictures. They were instructed to use the fewest number of moves possible, to only move one piece at a time, and to never place a larger disc on top of a smaller disc. This was repeated for 9 trials that increased in difficulty and the time provided to attempt to complete the trial. We recorded the time it took to complete each trial, the total number of moves required to complete each trial, and failures to complete the trial in the allotted time. These were used to compute achievement scores, according to published criterion (Tower Test Achievement Score). Achievement scores can range from 0 to 30.

#### Color-Word Interference Test (Stroop)

We also used the Stroop Test^45^ to assess executive functioning, specifically the ability to inhibit a prepotent response and to switch response sets. For the first (Color Naming) trial, participants were shown 5 rows of 10 color patches (e.g., a blue rectangle) and were asked to say each color as quickly and as accurately as possible. For the second (Word Reading) trial, they were shown 5 rows of 10 words (e.g., blue) printed in black ink and were asked to read the words as quickly and accurately as possible. For the third (Inhibition) trial, they were shown 5 rows of 10 color words printed in incongruent ink color (e.g., the word blue printed in red ink) and were asked to say the color of the ink the words were printed in as quickly and as accurately as possible. For the final (Switching) trial, they were shown 5 rows of 10 color words printed in incongruent ink colors. Some of the words were shown in boxes and others had no boxes. Participants were instructed to say the ink color the words were printed in unless they were in a box, in which case they should read the word and not say the ink color. The time to complete each trial was scored. Longer times indicate worse performance.

#### Demographic and Screening Questionnaire

Participants responded to a small series of items designed to assess demographic characteristics including their gender, age, ethnicity, education, and personal income. They were also administered a series of questions created to confirm their eligibility in the study, including prior diagnosis or treatment of psychological disorders, chronic medical or neurological disorders, head injuries, learning disabilities, prescription medication use, and illicit drug use.

#### Daily Sessions, Frequency, Age of Onset and Quantity of Cannabis Use Inventory (DFAQ-CU)

The DFAQ-CU^46^ is a self-report measure used to assess cannabis use patterns. It contains 33 items and six subscales that measure daily sessions, frequency, age of onset, quantity of flower, quantity of concentrates, and quantity of edibles typically consumed. Scores were computed by averaging the standardized (z transformed) items within each subscale, according to published scoring criterion^46^. The psychometric properties of this inventory have been previously established with the various subscales demonstrating good reliability, as well as predictive, concurrent, and discriminant validity^46^.

#### Alcohol Use Disorder Identification Test (AUDIT)

Problematic drinking was assessed using the AUDIT^33^, which consists of 10 items relating to alcohol use for which participants responded along a scale ranging from 0 to 4. Scores can therefore range from 0 to 40, with higher scores indicating greater alcohol consumption and higher symptomology of alcohol use disorder. Scores between 0 to 7 indicate low risk drinking, scores between 8 to 14 indicate hazardous or harmful drinking, and scores above 15 are indicative of alcohol dependence^33^. The AUDIT has demonstrated sound psychometric properties^47^.

#### Depression Anxiety Stress Scales (DASS-21)

Levels of depression, anxiety, and stress were assessed using the DASS-21^32^, which is a self-report inventory designed to measure these different, but related constructs. Participants were asked to respond to 21 different statements and indicate how much each has applied to them in the past week using a scale ranging from 0 (did not apply to me at all) to 3 (applied to me very much, or most of the time). Each of the three subscales contained seven statements, and scores for each subscale were computed by summing those items and then multiplying the sum by 2. As such, scores for each subscale could range from 0 to 42, with higher scores representing stronger endorsement of that dimension of distress. The DASS-21 and its subscales have been shown to possess good convergent and discriminant validity as well as high internal consistency across clinical and non-clinical samples^48,49^.

### Data analysis

Chi-square tests were used assess group differences in gender, ethnicity, and education. One-way analyses of variance were used to examine group differences in age, personal income, premorbid verbal IQ, depression, anxiety, stress, AUDIT scores, and THC in urine. Independent samples *t*-tests were used to compare cannabis use patterns in flower and concentrate users.

One-way analyses of covariance (ANCOVAs) were used to examine differences in cognitive test performance of the three groups, after controlling for differences in education, anxiety, and AUDIT scores. Partial eta-squared values were computed to establish effect sizes for these ANCOVAs, with values of 0.01 indicating a small sized effect, values of 0.06 indicating a medium sized effect and values of 0.14 and higher indicating a large sized effect^50^. Significant main effects were probed using Sidak post hoc tests. Cohen’s *d* values were computed to establish the effect size of significant post hoc comparisons, with values of 0.20 indicating a small sized effect, values of 0.50 indicating a medium sized effect, and values of 0.80 corresponding to a large sized effect^51^.

Two binary logistic regression analyses were conducted to examine group differences in performance on the episodic prospective memory test (due to binary scoring), after controlling for group differences in education, anxiety, and AUDIT scores. To explore differences between non-users and both groups of cannabis users, non-users were entered as the reference group. To separately explore differences between flower and concentrate users, flower users were entered as the reference group in a second analysis. Odds ratios are provided for significant effects as an index of effect sizes.

Pairwise deletion was used to handle the small amount (< 1%) of missing data. These data were missing at random due to invalid test administration occurring occasionally (e.g., a participant who was color-blind couldn’t complete the Stroop Test, a research assistant failed to administer a test, performance was not recorded properly, etc.) Because we conducted multiple comparisons, we report Benjamini–Hochberg adjusted *p* values that control for false discovery rate, which was set at 0.10^52^, in addition to standard (unadjusted) *p* values with alpha set to 0.05.

## Results

As shown in Table 2, there were significant main effects of group on performance on the episodic prospective memory test, the immediate and delayed recall trials of the verbal memory test (CVLT-II), the source memory test, and the incidental recall trial of the DSST. However, the main effects on the latter test were no longer significant after controlling for false discovery rate using Benjamini–Hochberg adjusted *p* values, and are therefore not considered further.

**Table 2:** Cognitive test performance.

Cognitive domain	Non-users	Flower users	Concentrate users	Inferential statistics	Adjusted *p*
Memory					
Episodic prospective memory	57.1%^a^	37.3%^b^	28.3%^b^	${\varvec{\upchi}}$^**2**^** = 9.62, *p* = 0.008, *R***^**2**^** = 0.07**	**0.08**
Habitual prospective memory	10.71 (0.49)	9.96 (0.62)	9.62 (0.61)	*F* = 0.87, *p* = 0.42, $\upeta$_p_^2^ = 0.01	0.76
Verbal memory–immediate	8.93 (0.22)^a^	7.66 (0.27)^b^	7.68 (0.27)^b^	***F* = 7.37, *p* = 0.001,** ${\varvec{\upeta}}$_**p**_^**2**^** = 0.08**	**0.02**
Verbal memory–delayed	10.05 (0.34)^a^	8.45 (0.42)^b^	8.61 (0.41)^b^	***F* = 4.64, *p* = 0.01,** ${\varvec{\upeta}}$_**p**_^**2**^** = 0.05**	**0.05**
Visuospatial memory–immediate	9.96 (0.20)	9.82 (0.25)	9.50 (0.24)	*F* = 1.00, *p* = 0.37,$\upeta$_p_^2^ = 0.01	0.74
Visuospatial memory–delayed	10.88 (0.19)	10.84 (0.24)	10.62 (0.23)	*F* = 0.37, *p* = 0.69, $\upeta$_p_^2^ = 0.005	0.81
Free recall–pictures/words	16.08 (0.80)	13.91 (1.00)	13.72 (0.99)	*F* = 1.80, *p* = 0.17, $\upeta$_p_^2^ = 0.02	0.42
Episodic recall	9.98 (0.29)^a^	9.33 (0.36)^ab^	8.94 (0.35)^b^	*F* = 2.24, *p* = 0.11, $\upeta$_p_^2^ = 0.03	0.31
Source memory	50.49 (0.74)^a^	49.52 (0.94)^ab^	46.88 (0.91)^b^	***F* = 4.39, *p* = 0.01,** ${\varvec{\upeta}}$_**p**_^**2**^** = 0.05**	**0.07**
Temporal-order memory	18.28 (0.54)	19.22 (0.67)	18.88 (0.66)	*F* = 0.53, *p* = 0.59, $\upeta$_p_^2^ = 0.006	0.84
Working memory	7.60 (0.26)	7.82 (0.33)	7.81 (0.32)	*F* = 0.16, *p* = 0.86, $\upeta$_p_^2^ = 0.002	0.86
Incidental recall	17.04 (0.55)^a^	14.92 (0.68)^a^	15.12 (0.67)^a^	***F* = 3.09, *p* = 0.048,** ${\varvec{\upeta}}$_**p**_^**2**^** = 0.04**	0.19
Psychomotor speed/attention					
DSST copy trial	63.71 (1.49)	62.32 (1.86)	64.16 (1.83)	*F* = 0.30, *p* = 0.74, $\upeta$_p_^2^ = 0.004	0.82
Selective attention	298.46 (5.85)	291.99 (7.17)	300.01 (7.04)	*F* = 0.40, *p* = 0.67, $\upeta$_p_^2^ = 0.005	0.84
Executive functioning					
Zoo map test profile score	3.07 (0.14)	2.79 (0.17)	2.84 (0.17)	*F* = 0.80, *p* = 0.45, $\upeta$_p_^2^ = 0.01	0.75
Tower test achievement score	18.54 (0.50)	18.37 (0.62)	18.03 (0.61)	*F* = 0.19, *p* = 0.83, $\upeta$_p_^2^ = 0.002	0.87
Stroop color naming time (s)	27.80 (0.65)	26.75 (0.79)	27.11 (0.78)	*F* = 0.47, *p* = 0.63, $\upeta$_p_^2^ = 0.006	0.84
Stroop word reading time (s)	20.54 (0.49)	20.00 (0.60)	20.94 (0.59)	*F* = 0.70, *p* = 0.50, $\upeta$_p_^2^ = 0.009	0.77
Stroop inhibition time (s)	45.72 (1.41)	41.38 (1.73)	45.82 (1.70)	*F* = 2.40, *p* = 0.09, $\upeta$_p_^2^ = 0.03	0.73
Stroop switching time (s)	54.30 (1.45)	52.03 (1.76)	55.46 (1.73)	*F* = 1.11, *p* = 0.33, $\upeta$_p_^2^ = 0.01	0.30

Values in reminder test row reflect the % of participants in each group who succeeded on the test. The remainder of the tests display marginal means (adjusted for education, anxiety, and AUDIT scores) with standard errors in parentheses. Values with no superscripts or shared superscripts do not differ significantly according to Sidak’s post hoc test, while those with different superscripts are significantly different at *p* < 0.05.

Significant values are in bold.

Results of Sidak post hoc tests comparing each group on these tests revealed that compared to non-users, both the flower users (*p* = 0.03, odds ratio = 2.60) and the concentrate users (*p* = 0.004, odds ratio = 4.10) performed significantly worse on the episodic prospective memory test. Relative to non-users, flower users (*p* = 0.005, *d* = 0.74) and concentrate users (*p* = 0.005, *d* = 0.73) performed significantly worse on the immediate recall trial of the verbal memory test. Similarly, flower users (*p* = 0.01, *d* = 0.62) and concentrate users (*p* = 0.003, *d* = 0.73) performed significantly worse than non-users on the delayed recall trial of the verbal memory test. The concentrate users (but not the flower users) further demonstrated significantly worse performance than the non-users on the source memory test (*p* = 0.02, *d* = 0.62). Of primary relevance, there were no significant differences between flower users and concentrate users on any of the cognitive tests (*p*s > 0.05). Ancillary sensitivity analyses comparing just the flower and concentrate users after controlling for group differences in flower quantity also revealed no significant group differences.

## Discussion

This is the first study to objectively compare cognitive test performance of non-users, flower users, and concentrate users under sober conditions. We found that, relative to non-users, both flower and concentrate users demonstrated poorer immediate and delayed verbal memory test performance, as well as episodic prospective memory test performance. Neither flower users nor concentrate users differed from non-users on measures of visuospatial memory, working memory, episodic memory, temporal-order memory, psychomotor speed/attention, or executive functioning. These findings provide limited support for the hypothesis that both concentrate and flower users would demonstrate worse cognitive test performance than non-users, particularly on tests of memory. That is, while between-group differences were observed for some (but not all) aspects of memory, no relevant differences were observed on the other domains of cognition assessed. We also found that concentrate users had worse source memory test performance relative to non-users. This was the only significant effect detected in concentrate users that was not detected in exclusive flower users. Crucially, however, none of the comparisons of flower and concentrate users revealed any significant group differences. As such, we found no support for our hypothesis that concentrate users would demonstrate worse cognitive test performance than flower users.

Findings from the present study indicating moderate sized detrimental effects of regular cannabis (flower or concentrate) use on verbal memory and prospective memory are consistent with previous studies demonstrating small-to-moderate-sized detrimental effects on tests of verbal memory^25,26^ and prospective memory^26,53,54^. Although effects of chronic cannabis use on executive functioning, attention, psychomotor speed, and decision-making tests have been previously reported^26,27^, we did not replicate them in this study. However, a recent meta-analysis of meta-analyses found that chronic effects in these domains are often smaller and less reliable than memory effects^26^ and as such we may have been underpowered to detect them.

To our knowledge, this is the first study to compare temporal-order memory and source memory test performance of chronic cannabis users and non-users. We observed group differences in source memory but not temporal-order memory, which extends upon findings that *acute* intoxication from high-potency cannabis (but not low-potency cannabis^55,56^) produces source memory decrements while sparing temporal-order memory^17^ These findings are important as source and temporal-order memory are better predictors of problems with everyday life functioning than more traditional measures of free recall^40^.

Our finding that concentrate users did not perform significantly worse than flower users on any cognitive test is somewhat surprising given past literature indicating concentrate users perceive greater risk of developing problems with cognition^8^ and subjectively report poorer memory and attention when using concentrates compared to flower^20^. Nevertheless, our findings are consistent with the predominantly null results of the three *acute* studies examining cognitive test performance following participants’ self-administration of flower versus concentrates^17,18,28^. Specifically, those studies revealed no significant differences in flower and concentrate users' performance on numerous measures of memory (verbal, episodic, working, prospective, false, and temporal-order memory), psychomotor speed, and non-normative decision-making. Despite the administration of several cognitive tests across these three studies, collectively, concentrate users were found to perform worse than flower users on only one test of inhibitory control when collapsing across all assessment periods, including before and after use^18^. These predominantly null *acute* effects may reflect the fact that concentrate users take fewer hits of the higher potency form of the drug^17^, and by doing so report comparable levels of intoxication when under the influence of concentrates versus flower^17,18^. Similarly, the lack of group differences in the *chronic* use of concentrates versus flower in the present study may also simply reflect the tendency for concentrate users to use lower quantities of the higher-potency version of the drug, thereby exposing them to similar levels of THC.

In a similar vein, flower and concentrate users in the present study did not differ in terms of age of onset of cannabis use, duration of use, daily cannabis use session, self-reported frequency of cannabis use or urine THC levels, but concentrate users did report using larger quantities of cannabis flower than exclusive flower users. Although not the focus of this study, these findings are largely inconsistent with previously reported differences between flower and concentrate users’ frequency of cannabis use^20,22^, age of onset of cannabis use^23^, and plasma THC concentrations^18^, but potentially supportive of prior findings indicating that concentrate use is associated with higher levels of tolerance than cannabis flower use^19^. Specifically, the latter finding could reflect that concentrate users are somewhat tolerant to the intoxicating effects of THC and may therefore require higher quantities of cannabis flower to achieve similar levels of intoxication as would be achieved via concentrate use. Further, we note, that the prior study that found differences in plasma THC concentration used high-performance liquid chromatography/mass spectroscopy to examine THC and its metabolites in plasma^18^, while we relied on a less sensitive commercially available measure of THC in urine limited to only six levels of detection (0 ng/ml, 25 ng/ml, 50 ng/ml, 150 ng/ml, 300 ng/ml, 600 ng/ml). We found that, on average, THC concentrations in both concentrate and flower users exceeded the 300 ng/ml threshold, but the threshold for the next level of concentration was twice as high (600 ng/ml) and therefore subtle group differences in THC concentration may have escaped our detection.

Chronic concentrate use could potentially be associated with worse cognitive test performance only when used by individuals showing elevated risk factors (e.g., more severe symptoms of cannabis use disorder, severe symptoms of psychological disorders, higher frequency of *concentrate* use, lack of self-titration, early age of onset, etc.). While these questions are outside the scope of the present study, future research should examine whether similar null findings are observed in higher risk individuals. Regardless, results of the present study serve as an important foundational steppingstone by demonstrating that regular use of concentrates does not inevitably produce more cognitive impairment than exclusive use of cannabis flower.

## Limitations

The present study focused on healthy adults, excluding individuals over the age of 39 as well as individuals with psychiatric, neurological, or serious medical conditions and those who recently used illicit drugs. Given past research shows that concentrate users are more likely to use other drugs^20,23^ and to be diagnosed with a psychological disorder, including depression and anxiety^20,21^, our exclusion of these individuals could have diminished sensitivity to detect group differences in cognition and/or led to a biased sample who are less representative of concentrate users. Nevertheless, these exclusion criteria were deemed important as older age, these conditions, and recent use of illicit drugs can be detrimental to cognition. Our objective measure of cannabis use was not highly sensitive and we relied on self-reports of illicit drug use and psychiatric/medical symptoms rather than objective drug tests and full psychiatric/medical interviews. While 18 people disclosed recent use of illicit substances, 30 reported medical/neurological conditions, and 20 people disclosed a psychological diagnosis during the second screening (at the end of the testing session), and were therefore excluded from analyses, it is possible that some participants were less honest and evaded detection using these methods.

Further, nearly all participants in the concentrate user group reported also using flower. Although this is consistent with findings that concentrate users often use both flower and concentrates, whereas only a small percentage are exclusive concentrate users^10^, implications of using a mixed group must be considered. Use of an exclusive concentrate user group and an exclusive flower user group would clarify differences in chronic effects of these two product types per se. In contrast, use of a mixed group is relevant for understanding how chronic concentrate use influences cognition in the manner in which they are commonly used in the broader population of cannabis users (i.e., in conjunction with flower). That is, while the effects of exclusive concentrate use may differ than the results reported herein, such effects only generalize to a relatively small portion of cannabis users. Nonetheless, the chronic and acute effects of exclusive concentrate use should be considered in future research to isolate specific effects of concentrates relative to flower.

Finally, although we measured types of products used, quantities of these products typically used, and frequency of cannabis use overall, we did not measure frequency of concentrate use or flower use specifically. Future research should examine whether cognitive performance varies depending upon the relative frequency of concentrate to flower use. Similarly, the literature has not properly addressed whether concentrates and flower differ with respect to the ratio of THC to CBD content. Given mixed evidence of modulating effects of CBD on cognition^17,55,57,58^, future research should measure and consider ratios of THC to CBD in products used (although these would likely vary across cannabis use sessions).

Finally, the present study is limited by its non-experimental, cross-sectional design. While it would be unethical to randomly assign individuals to regularly use concentrates versus flower, longitudinal research examining changes in cognition from before to after initiating regular use of flower versus concentrates may be illuminating (but challenging).

## Conclusions

The state-level legal cannabis market has introduced a diverse array of new high-potency cannabis products to consumers, including extremely high-potency concentrates that are rapidly growing in popularity^5^. This has raised concerns as higher concentrations of THC are believed to magnify the harms of cannabis use, including increased risks of dependence^12^, psychosis^13,14^, and cognitive impairment^15,16^. Nevertheless, the federal status of cannabis as a Schedule 1 drug has histrorically limited researchers’ ability to study high-potency forms of the drug and consequently we know very little about the objective effects of high-potency cannabis products. Results from the present study indicate that regular use of cannabis flower or concentrates is associated with worse verbal memory and episodic prospective memory, but that individuals who use concentrates regularly are no more cognitively impacted than those who exclusively use flower under sober conditions. This lack of difference likely reflects a tendency for people to self-titrate their use of concentrates. Nevertheless, future research should replicate and expand on our findings to contribute to a well-rounded literature regarding the chronic and acute effects of these under investigated, extremely high-potency products.

## Data Availability

Data are available upon request to Carrie Cuttler at carrie.cuttler@wsu.edu.
